# Sex differences in response to maximal exercise stress test in trained adolescents

**DOI:** 10.1186/1471-2431-12-127

**Published:** 2012-08-20

**Authors:** Åsa Fomin, Mattias Ahlstrand, Helena Gyllenhammar Schill, Lars H Lund, Marcus Ståhlberg, Aristomenis Manouras, Anders Gabrielsen

**Affiliations:** 1 Department of Cardiology, Karolinska Institutet, Karolinska University Hospital, N1:05, SE-17176, Stockholm, Sweden

**Keywords:** Adolescent, Sex, Body composition, Exercise stress test, ECG, Blood pressure, Peak VO_2_, Ventilation, Lactate

## Abstract

**Background:**

Sex comparisons between girls and boys in response to exercise in trained adolescents are missing and we investigated similarities and differences as a basis for clinical interpretation and guidance.

**Methods:**

A total of 24 adolescent females and 27 adolescent males aged 13–19 years underwent a maximal bicycle exercise stress test with measurement of cardiovascular variables, cardiac output, lung volumes, metabolic factors/lactate concentrations and breath-by-breath monitoring of ventilation, and determination of peak VO_2_.

**Results:**

Maximum heart rate was similar in females (191 ± 9 bpm) and males (194 ± 7 bpm), cardiac index at maximum exercise was lower in females (7.0 ± 1.0 l/min/m^2^) than in males (8.3 ± 1.4 l/min/m^2^, P < 0.05). Metabolic responses and RQ at maximum exercise were similar (females: 1.04 ± 0.06 vs. males: 1.05 ± 0.05). Peak VO_2_ was lower in females (2.37 ± 0.34 l/min) than in males (3.38 ± 0.49 l/min, P < 0.05). When peak VO_2_ was normalized to leg muscle mass sex differences disappeared (females: 161 ± 21 ml/min/kg vs. males: 170 ± 23 ml/min/kg). The increase in cardiac index during exercise is the key factor responsible for the greater peak VO_2_ in adolescent boys compared to girls.

**Conclusions:**

Differences in peak VO_2_ in adolescent boys and girls disappear when peak VO_2_ is normalized to estimated leg muscle mass and therefore provide a tool to conduct individual and intersex comparisons of fitness when evaluating adolescent athletes in aerobic sports.

## Background

Physical activity and training is important for initiating and sustaining cardiovascular health. As such, encouragement from childhood and the possibility to participate in sports activity is a major health issue which must be sustained. Reaching adolescence, however, increasing expectations and competitive demands have gradually emerged as an important aspect of recreational sports in the young. During the past decades there has been a gradual increase in the number of teenagers participating in competitive sports and the number of female athletes has increased in aerobic team sports [1,2] – in Sweden in particular in soccer, floor-ball and ice-hockey [3]. Correct and adequate planning of physical and exercise training is instrumental in providing the young athlete with a sustained career, increase endurance and enhance musculo-skeletal stability and avoiding injuries, fatigue or physical scarring.

Adolescence is the age interval at which the young sport competitor starts building local and national team careers, prompting coaches and physical trainers to interpret test results and understand and take into account possible sex-specific characteristics. At the moment, however, detailed knowledge about sex-related differences in adolescent exercise physiology is limited. Maximal oxygen uptake has been extensively investigated in children [4-7] and adults [8], but clinical context sex comparisons in the adolescent age-group 13 to 19 years of age in trained subjects are missing.

This impedes our interpretation of the cardiovascular and respiratory responses to exercise in adolescent girls compared to boys and limits our guidance in exercise programs in adolescents in competitive sports. It is essential to acknowledge possible differences between male and female participants which, if present, must be taken into account when planning and performing physical exercise programs for the adolescent athlete. Differences between male and female participants may prompt the development of sex-specific programs, and be valuable when evaluating training levels and differentiating pathology from normality in youth sports.

Because clinical performance reference data are missing we performed a detailed comparative characterization of cardiovascular, respiratory and metabolic responses to maximum exercise in aerobically trained adolescent boys and girls, with the aim being to identify similarities and differences as a basis for clinical interpretation and guidance.

## Results

### Subjects and physical characteristics

One female participant was diagnosed with a Wolf-Parkinson-White type pre-excitation but was included in the study as she was completely asymptomatic. She was referred for cardiac electrophysiological evaluation. Her other exams and test results, beside the ECG, were completely normal. All other subjects had a normal medical history, a normal physical exam, and normal pre-study echo-cardiographic exam.

All exercise trials were terminated due to general fatigue with maximum leg fatigue, whereas no trial was stopped prematurely because of dyspnea, chest pain or cardiac arrhythmias.

Age was similar in males and females, the males being taller and heavier but with similar body mass index compared to female athletes (Table 1). In addition, expected differences in segmental body composition, hemoglobin and creatinine were observed (Table 1). Female subjects participated in formal training and/or exercise a little less than did male subjects (Table 1). When comparing baseline ECG characteristics only QTc interval differed between female and male subjects (Table 1). No differences in plasma concentrations of Na^+^ and K^+^, platelet- or leukocyte counts were observed (data not shown).

**Table 1:** Baseline characteristics of the study subjects

	**Females (n = 24)**	**Males (n = 27)**	**P-value**
Age (years)	16.5 ± 1.8	17.0 ± 1.3	0.26
**Height (cm)**	**168 ± 5**	**179 ± 6**	**P < 0.001**
**Weight (kg)**	**61.3 ± 9.2**	**69.7 ± 9.1**	**P = 0.002**
BMI (kg/m^2^)	21.6 ± 2.8	21.7 ± 2.7	P = 0.84
**Training pr week (hours)**	**7.4 ± 1.6**	**8.8 ± 1.3**	**P = 0.002**
**Total body fat (kg/%)**	**15.1 ± 5.3/23.1 ± 6.5**	**8.9 ± 4.2/12.7 ± 5.1**	**P < 0.001**
**Total body fat free mass (kg)**	**46.6 ± 5.3**	**60.7 ± 7.7**	**P < 0.001**
**Total leg muscle mass (kg)**	**15.0 ± 1.9**	**20.0 ± 2.7**	**P < 0.001**
**Hemoglobin (g/l)**	**132 ± 6**	**147 ± 8**	**P < 0.001**
**Creatinine (μmol/l)**	**65 ± 10**	**78 ± 9**	**P < 0.001**
**Resting ECG parameters**			
PR interval (msec)	149 ± 21 (149 ± 21; N = 23)	144 ± 14	P = 0.39
QRS duration (msec)	95 ± 20 (92 ± 7.6; N = 23)	99 ± 9.9	P = 0.43
**QTc (msec)**	**440 ± 36 (435 ± 25; N = 23)**	**417 ± 20**	**P = 0.007**
P axis (°)	52 ± 26 (52 ± 26; N = 23)	50 ± 28	P = 0.79
QRS axis (°)	65 ± 38 (70 ± 29; N = 23)	80 ± 24	P = 0.10
T axis (°)	59 ± 47 (50 ± 17; N = 23)	54 ± 17	P = 0.57

Values are expressed as mean ± SD. Rows in bold denotes a statistical significant difference (P < 0.05) between females and males. P-values represent values after correction for multiple testing. In the section reporting ECG parameters female data is presented with (N = 24) and without (N = 23; parentheses) inclusion of the subject with WPW-type pre-excitation.

### Resting cardiopulmonary and metabolic characteristics

**Figure 1:**
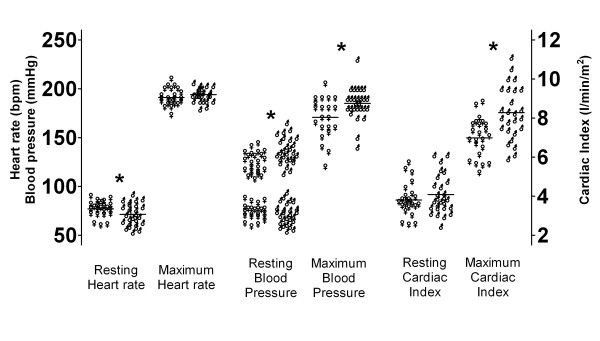
**Heart rate, blood pressure, and cardiac index during baseline and at maximum exercise in boys and girls.** Recordings of heart rate, blood pressure, and cardiac index during baseline and at maximum exercise in female (♀, N = 24; cardiac index measurements N = 23) and male (♂, N = 27; cardiac index measurements N = 26) subjects. During exercise only systolic blood pressure was recorded. *) denotes statistical significant difference (p < 0.05 by unpaired t-test with correction for multiple comparisons) between female and male subjects with regard to resting heart rate, resting and maximum systolic blood pressure, and maximum cardiac index.

Female subjects exhibited a lower systolic blood pressure (124 ± 11 mmHg; mean ± SD) and higher resting heart rate (77 ± 8 bpm) compared to male participants (137 ± 13 mmHg and 71 ± 11 bpm, respectively; P < 0.05), whereas diastolic blood pressure (females: 74 ± 8 mmHg vs. males: 72 ± 11 mmHg) and resting cardiac index (females: 3.8 ± 0.8 l/min/m^2^ vs. males: 4.1 ± 1.0 l/min/m^2^) did not differ between sexes (Figure 1). Ventilated lung volumes at rest were lower in females than in males (P < 0.05, Table 2). There were no differences in metabolic variables; blood lactate concentration, blood pH, blood base excess, and blood HCO_3_^-^ at rest (Table 2). At 60 watt (initial step) exercise absolute VCO_2_ were slightly lower in females (0.55 ± 0.13 l/min) than in males (0.65 ± 0.11 l/min, p < 0.05), whereas VO_2_ did not differ (females: 0.79 ± 0.12 l/min vs. males: 0.85 ± 0.15 l/min) leading to a minor difference in RQ (females: 0.71 ± 0.12 vs. males: 0.77 ± 0.08, P < 0.05). Ventilation volumes at this initial step of exercise were similar comparing females and males (Table 2).

**Table 2:** Response to exercise of selected ventilatory and metabolic variables in the study subjects

	**Females (N = 24)**		**Males (N = 27)**	
	**Before exercise/rest**	**Maximum/after exercise**	**Before exercise/rest**	**Maximum/after exercise**
**Ventilation and lung volumes**				
Ventilation (l/min)	#23.5 ± 5.7; N = 22	83.5 ± 12.3; N = 23 *	#24.9 ± 5.1	110.7 ± 16.4 *†
Normalized ventilation (l/min/kg)	#0.39 ± 0.08; N = 22	1.38 ± 0.20; N = 23 *	#0.36 ± 0.09	1.61 ± 0.26 *†
Ventilated lung volume (l)	2.58 ± 0.56	3.55 ± 0.54; N = 19 *	3.78 ± 0.66 †	4.82 ± 1.06; N = 25 *†
Normalized ventilated lung volume (l/kg)	0.043 ± 0.012	0.060 ± 0.012; N = 19 *	0.055 ± 0.011 †	0.071 ± 0.016; N = 25 *†
**Metabolic factors**				
Blood lactate (mmol/l)	1.60 ± 0.31;N = 22	11.63 ± 2.47;N = 23 *	1.70 ± 0.50	13.03 ± 3.22 *†
Blood pH	7.356 ± 0.030;N = 22	7.254 ± 0.049;N = 23 *	7.354 ± 0.027	7.248 ± 0.048 *
Blood base-excess (mmol/l)	0.76 ± 1.07;N = 22	−9.77 ± 2.87;N = 23 *	1.34 ± 0.89	−10.18 ± 2.93 *
Blood HCO_3_^-^ (mmol/l)	24.4 ± 1.0;N = 22	16.6 ± 2.0;N = 23 *	24.9 ± 0.8	16.5 ± 2.0 *

Values are expressed as mean ± SD. Default number of subjects are N = 24 for female and N = 27 for male, respectively, otherwise the specific number of subjects included in the analysis is noted. #) denotes that ventilation and lung volume measurements were performed with “Before exercise/rest” values recorded during the initial stable phase of exercise at 60 watt and compared to recordings made during maximum exercise. Metabolic factors were measured at rest before start of exercise and 10 min following the end of maximal exercise. *) denotes a statistical significant difference (P < 0.05, by ANOVA) comparing corresponding variable “Before exercise/rest” with “Maximum/after exercise”. †) denotes a statistical significant difference (P < 0.05, by ANOVA) comparing female and male subjects.

### Cardiopulmonary and metabolic responses to exercise

**Figure 2:**
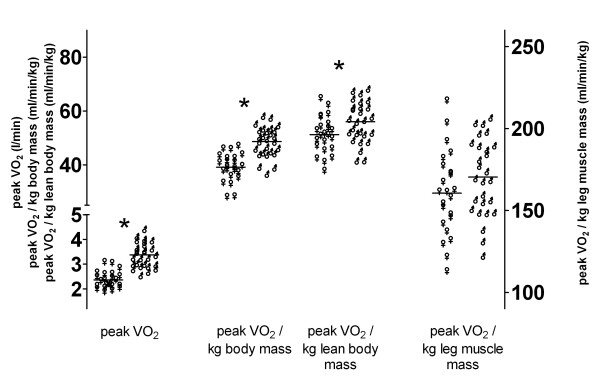
**Oxygen uptake in boys and girls.** Peak oxygen uptake (peak VO_2_, l/min) and peak oxygen uptake normalized to total body mass (ml/min/kg), normalized to estimated fat free mass (ml/min/kg lean body weight), and normalized to estimated total leg muscle mass (ml/min/kg leg muscle) in female (♀, N = 23) and male (♂, N = 27) subjects. *) denotes statistical significant difference (p < 0.05 by unpaired t-test with correction for multiple comparisons) between female and male subjects with regard to peak VO_2_, peak VO_2_ normalized to total body mass and estimated lean body mass, but not comparing peak VO_2_ normalized to estimated leg muscle mass.

Maximum systolic blood pressure during exercise was lower in female (171 ± 20 mmHg) than in male subjects (185 ± 17 mmHg, p < 0.05), whereas maximum heart rate was similar (females: 191 ± 9 bpm vs. males 194 ± 7 bpm, Figure 1). Cardiac index at maximum exercise was lower in females (7.0 ± 1.0 l/min/m^2^) than in males (8.3 ± 1.4 l/min/m^2^, P < 0.05, Figure 1). Ventilation and ventilated lung volume at maximum exercise were significantly lower in females than in males (P < 0.05, Table 2), whereas metabolic responses to maximum exercise were very similar with only a minor incremental increase in blood lactate levels in males compared to females and without differences in blood pH, blood base excess, or blood HCO_3_^-^ (Table 2). Peak VCO_2_ were lower in females (2.45 ± 0.36 l/min) than in males (3.50 ± 0.51 l/min) but was matched to peak VO_2_ in a similar manner in both sexes as RQ at maximum exercise was similar (females: 1.04 ± 0.06 vs. males: 1.05 ± 0.05). Absolute peak VO_2_, peak VO_2_ normalized to total body mass, and peak VO_2_ normalized to lean body mass were lower in females (2.37 ± 0.34 l/min; 39.1 ± 5.1 ml/min/kg; 51.2 ± 6.8 ml/min/kg, respectively) compared to males (3.38 ± 0.49 l/min; 48.7 ± 5.5 ml/min/kg; 56.0 ± 7.6 ml/min/kg, respectively, P < 0.05; Figure 2), but when peak VO_2_ was normalized to leg muscle mass sex differences disappeared (females: 161 ± 21 ml/min/kg vs. males: 170 ± 23 ml/min/kg; Figure 2). Normalization of peak VO_2_ to estimated leg muscle mass, when compared to normalization to total body mass/lean body mass had an impact on the classification rank of training-level in girls. In our group of girls, 25% (N = 6) were reclassified and shifted from the half of subjects with peak VO_2_ below the median to the group above the median when normalization to total body mass/lean body mass was compared to peak VO_2_ normalization to estimated leg muscle mass. This impact of normalization on classification rank of training-level was less apparent in boys (2 subjects reclassified) – mainly because their estimated fat mass in legs exhibits very low levels with low variation.

## Discussion

In this investigation we demonstrate expected sex differences in the response to a maximal bicycle stress exercise test in trained adolescents [8] and that the cardiovascular, respiratory and metabolic differences are essentially similar to those observed in adults [8-10]. We also demonstrate that maximum heart rate, systemic lactate levels, and RQ are similar in adolescent boys and girls, which indicates that maximum performance is associated with the same metabolic adaptation irrespective of sex. However, we observe that the normally observed difference in peak VO_2_ per kilogram body mass comparing boys and girls disappears when peak VO_2_ is normalized to leg muscle mass only. This finding suggests that, when applied to aerobically trained subjects, there is a close relationship between leg muscle mass and peak VO_2_ and that peak VO_2_ normalized to leg muscle mass serves as a guiding measure when evaluating the individual level of aerobic performance and comparing the degree of fitness between boys and girls in aerobic sports.

The cardiopulmonary and metabolic response to a maximal bout of exercise has been well studied in adults and differences between sexes have been previously described [8,10]. However, when we were to start an evaluation program of the training levels and physical performances of adolescent female floor-ball players, it was unclear which peak VO_2_ levels, and metabolic differences and/or similarities should be accounted for by sex when comparing boys and girls in this category of dynamically exercising [11] adolescents. A number of studies have evaluated VO_2_max in children and adolescents, and generally it appears that in children below 12 years of age VO_2_max is slightly higher in boys than in girls, whereas differences begin to expand during adolescence [5,7,8,12,13]. Reaching adolescence, however, clinical and experimental data regarding ventilation, metabolism and hemodynamics are scarce and interpretation of test results and referencing is difficult.

In this investigation, we demonstrate lower resting and working blood pressures and higher resting heart rates in females, resting ECG patterns are similar except for QTc which is longer in females. Furthermore, aerobically trained adolescent girls and boys increase their heart rate and lactate levels to similar limits, being slightly higher in boys but without any detectable differences abroad the age-span investigated, in response to an aerobic maximal stress exercise test. Females and males exhibit similar maximum heart rates, which translate into a greater cardiac index in boys, being the one key factor leading to a greater peak VO_2_. The metabolic compensation, however, demonstrated a very similar adaption as pH, HCO_3_^-^, and the respiratory ratio (RQ) exhibit identical responses. Taken together, a pattern very similar to that observed in aerobically trained young adults [8,9].

In terms of peak VO_2_, we observed absolute values similar to those previously reported in this age group [5] with a difference between females and males when comparing absolute peak VO_2_, peak VO_2_ normalized per kilogram total body mass, or lean body mass. When normalizing to estimated leg muscle mass, however, girls and boys demonstrated similar peak VO_2_. We take this to demonstrate that peak VO_2_ is balanced against leg muscle mass in aerobically trained adolescent girls and boys. This finding is in line with previous observations in adults [14-16] but not in children [7,13,17]. In the study by Sunnegårdh & Bratteby [13], however, females exhibited lower levels of physical activity, as a contributory explanation for a lower VO_2_max, and a possible sex difference in VO_2_max normalized to estimated lean leg volume [15], in other words differences may be much smaller in trained compared to untrained subjects. When interpreting their data in relation to observations in children by Davies et al. [18] it appears that VO_2_max normalized to lean leg volume/leg muscle mass would be similar in boys and girls around 15–16 years of age [13] and older. Integrating these previous findings with observations from this investigation we propose that peak VO_2_ normalized to leg muscle mass is an acceptable clinical indicator to evaluate individual aerobic performance in relation to body composition and perform inter-sex comparisons in aerobically trained adolescents. In addition, the normalization of peak VO_2_ to estimated leg muscle mass, when compared to normalization to total body mass/lean body mass has an impact on the classification rank of training-level in girls, but much less so in boys.

Finally, when comparing the maximal exercise stress tests between girls and boys, we made the subjective observation that boys exhibit an operator-perceived degree of exercise stress that is more pronounced than in girls. Accordingly, the operator, without taking into account physiological measurements during the test would feel that the boys put in a greater effort than the girls, and that the girls *appeared* not to reach the point of total exertion. Examining the physiological responses of maximum heart rate or blood lactate levels, however, demonstrate that the girls are at a similar point of exertion as is the boys. We believe that this “wrong” perception that girls appear not to reach full exertion during testing is mainly caused by differences in ventilation volumes; with greater volumes and panting in boys (Table 2), leading to psychological perception of higher effort. Obviously, such a perceptive bias is important to note when working with exercise training in adolescent girls.

## Conclusions

In conclusion, we demonstrate that the main factors to realize when working with aerobic training programs in female adolescents are: 1) resting blood pressure is lower, resting heart rate is higher and the ECG exhibits longer QTc interval when compared to males; 2) in response to a maximal bicycle exercise stress test maximum heart rate, maximum blood lactate levels and metabolic compensation are similar in adolescent females and males; 3) the increase in cardiac index during exercise is the key factor responsible for the greater absolute peak VO_2_ in adolescent boys compared to girls; 4) observed difference in peak VO_2_ per kilogram body mass comparing boys and girls disappears when peak VO_2_ is normalized to leg muscle mass only. This finding suggests that peak VO_2_ normalized to leg muscle mass could be a useful measure when comparing the degree of fitness between adolescent boys and girls in aerobic sports; and 5) there is a subjective operator-perceived impression that girls do not reach full exertion during testing, but physiological measurements clearly show a level of exertion similar to that of boys.

## Methods

### Study subjects

A total of 24 adolescent female and 27 adolescent male floor-ball players, i.e. an activity with a high dynamic (aerobic) component and a low/moderate static (anaerobic) component [11], were recruited from teams of a local floor ball club and investigated according to the protocol described below. All subjects and their parents gave written informed consent to participation following comprehensive oral and written information about the study protocol and the aims of the study. The investigation was approved by the ethical committee of Northern Stockholm (Young Athlete Heart Study, 2009/1246-31/1) and conformed to the principles set forth in the declaration of Helsinki.

### Study protocol

The subject reported to the study room and underwent a health interview regarding family and personal medical history and a physical exam. The subject’s height, weight and results of segmental body impedance were recorded. A standardized cardiac ultrasound examination was then performed.

Thereafter, a peripheral venous catheter was inserted into an ante-cubital vein and basal blood samples drawn. A baseline supine resting 12-lead ECG and blood pressure was recorded and the subject thereafter placed on the exercise bicycle instrumented with 12-lead exercise ECG electrodes, blood pressure measurement cuff, and a mouth-piece and nose-clip so that breath-by-breath ventilation, O_2_ and CO_2_ gas-exchange, and pulmonary blood flow by inert gas rebreathing technique could be measured.

The subjects then performed the exercise bicycle test using the same exercise protocol, starting at basal 60 watts load with 20 watts increments per minute, in both females and males. The subjects performed the test until self-experienced total exertion, and maximum stress of dyspnea, leg fatigue, and chest pain recorded by means of a modified visual-analog Borg scale ranging from 0 (no perceived stress) to 20 (maximum perceivable stress) [19,20]. Following the exercise test the subjects were de-instrumented and immediately placed in the supine position for recovery and post-stress blood samples were collected and the protocol terminated.

### Measurements

#### Total body impedance

Subject body composition, i.e. body fat, water, and muscle composition, including segmental body and limb analysis of fat and muscle masses were measured by bioimpedance using a Tanita Body Composition Analyser BC 418MA device (Tanita, Helsinge, Denmark) [21]. Bioimpedance provides an acceptable estimate and was chosen over the more accurate dual energy x-ray absorptiometry or magnetic resonance imaging because they were considered not to be ethically feasible in this investigation.

#### Blood tests

Before the exercise test baseline blood samples were collected for the analysis of blood chemistry including red blood cell count, hematocrit, total hemoglobin, mean corpuscular volume, mean corpuscular hemoglobin concentration, leukocyte count, platelet count, Na, K, creatinine, and forearm venous blood gas chemistry (ABL 700, Radiometer, Copenhagen, Denmark) for metabolic assessment (base-excess, HCO_3_^-^, pH) and blood lactate concentration [22]. An additional forearm venous blood gas chemistry sample was drawn 10 min following maximal exercise stress for post-stress analysis.

#### Echo-cardiography

All subjects underwent a pre-study echo-cardiographic exam. A commercially available ultrasound apparatus (Vivid i, GE Vingmed, Horten, Norway) was employed for echo-cardiographic examinations using a standard phased 2.5 MHz multi-frequency transducer. The examinations were performed with the participant lying at the left lateral recumbent position at the end of expiration and the images acquired according to the recommendations of the European Society of Echocardiography from the parasternal and apical views [23].

#### Blood pressure

Resting blood pressure was recorded in the supine position using a semi-automatic blood pressure recording device (Omron 705IT, Omron Healthcare). During exercise systolic blood pressures were recorded using a blood pressure cuff and an ultrasound Doppler flow measurement probe positioned over the radial artery.

#### Exercise stress bicycle setup and ECG-recording

The stress test was performed on a Rodby Ergometer Bike RE990 stress test bicycle with a pre-loaded protocol (60 watt start / 20 watt increment/min) and an integrated standard 12-lead ECG recording (GE Case, GE Healthcare, Sweden). The 12-lead ECG was recorded at supine rest and during the whole exercise protocol to evaluate ST-segment deviations, arrhythmia and maximum heart rate.

#### Ventilation, gas-exchange, pulmonary blood flow and cardiac output

During the exercise stress test the subjects were connected to an Innocor® device (Innovision, Odense, Denmark) through a breathing mouth piece with continuous inspiratory and expiratory gas sampling for analysis and flow measurement. Mouth-circuit breathing was secured by the subject wearing a tight nose-clip to prevent nasal ventilation. Ventilation, O_2_, and CO_2_ exchange was measured on a breath-by-breath basis and stored on the hard drive for subsequent analysis. Peak VO_2_ was derived as the average VO_2_ obtained during the final 30 seconds of exercise and normalized to total body weight, lean body weight, i.e. fat free mass, and estimated leg muscle mass respectively. Using the same Innocor® device, pulmonary blood flow was measured at baseline with the subject seated on the bicycle ergometer and at maximal exercise using the inert gas rebreathing technique. To measure pulmonary blood flow the subject performed 30 seconds of closed circuit rebreathing from a bag containing a gas mixture of 50% O_2_, 5% N_2_O (soluble gas), 1% SF_6_ (insoluble gas) in N_2_ diluted with ambient room air. The rebreathing bag volume was set individually in each subject as 30% above the expected tidal volume. Pulmonary blood flows and ventilated lung volumes were calculated using standard formulas as a part of the Innocor® software (Innovision, Odense, Denmark) [24,25]. Pulmonary blood flow was assumed to equal cardiac output as there was no pulmonary shunting of blood, and cardiac index was calculated from cardiac output according to standard formula.

### Statistical analysis

Statistical analysis was performed using the Statistica v 10 (Statsoft Inc., Tulsa, USA). Unpaired t-test with correction (Newman-Keuls) for multiple comparisons was used to detect differences in selected variables between female and male subjects. Primary variables pre-defined for analysis were: Anthropometric data, peak VO_2_, peak VO_2_ per kilogram body mass, peak VO_2_ per kilogram lean body mass, peak VO_2_ per kilogram leg muscle mass, maximum heart rate and blood pressure during exercise, ventilation, ventilated lung volumes, ventilated lung volumes per kilogram body mass, changes in metabolic; i.e. base-excess and pH and lactate levels. ANOVA analysis with post-hoc testing corrected for multiple comparisons (Newman-Keuls) was used to analyze responses to exercise and effect of sex. P < 0,05 was considered statistical significant.

## Competing interests

The authors declare that they have no competing interests.

## Authors’ contributions

ÅF: Conceived, planned and conducted the study, contributed to data analysis and intellectual contents of the manuscript. MA: Conceived, planned and conducted the study, contributed to data analysis and intellectual contents of the manuscript. HGS: Participated in the conduct of the study, and performed data analysis. Input on the intellectual contents of the manuscript. LHL: Contributed to planning of the study and contributed to intellectual contents of the manuscript. MS: Participated in the conduct of the study, and performed data analysis. Input on the intellectual contents of the manuscript. AM: Conceived, planned and conducted the study, performed data analysis and wrote the manuscript. AG: Conceived, planned and conducted the study, performed data analysis and wrote the manuscript. All authors read and approved the final manuscript.

## Authors’ information

Åsa Fomin and Mattias Ahlstrand were equal first author contributors. Aristomenis Manouras and Anders Gabrielsen were senior author contributors.

## Pre-publication history

The pre-publication history for this paper can be accessed here:

http://www.biomedcentral.com/1471-2431/12/127/prepub
